# A Review of the Clinical Applications of Artificial Intelligence in Abdominal Imaging

**DOI:** 10.3390/diagnostics13182889

**Published:** 2023-09-08

**Authors:** Benjamin M. Mervak, Jessica G. Fried, Ashish P. Wasnik

**Affiliations:** Department of Radiology, University of Michigan—Michigan Medicine, 1500 E. Medical Center Dr., Ann Arbor, MI 48109, USA; bmervak@med.umich.edu (B.M.M.); jefried@med.umich.edu (J.G.F.)

**Keywords:** artificial intelligence, machine learning, deep learning, radiology, abdominal imaging, body imaging, MRI, CT, US

## Abstract

Artificial intelligence (AI) has been a topic of substantial interest for radiologists in recent years. Although many of the first clinical applications were in the neuro, cardiothoracic, and breast imaging subspecialties, the number of investigated and real-world applications of body imaging has been increasing, with more than 30 FDA-approved algorithms now available for applications in the abdomen and pelvis. In this manuscript, we explore some of the fundamentals of artificial intelligence and machine learning, review major functions that AI algorithms may perform, introduce current and potential future applications of AI in abdominal imaging, provide a basic understanding of the pathways by which AI algorithms can receive FDA approval, and explore some of the challenges with the implementation of AI in clinical practice.

## 1. Introduction

The past decade has seen a rapid acceleration in interest and hype regarding artificial intelligence (AI), machine learning (ML), deep learning (DL), and potential applications in healthcare. As a gross measure, PubMed results on AI or ML increased more than 20-fold from roughly 600 scientific papers in 2009 to over 12,500 papers in 2019 [1]. Subsequently, the number of algorithms receiving approval from the United States Food and Drug Administration (FDA) under the umbrella of “software as a medical device” (SaMD) has also rapidly increased, from 7 approved algorithms in early 2016 to more than 200 in early 2023.

As AI and ML algorithms represent adaptable mathematical tools rather than a single static instrument, they have been heralded in widespread applications within radiology departments, with proposed uses including image quality control, augmentation and prioritization of workflows and worklists, segmentation of organs and lesions, detection of incidental findings, and assisting with classification [2]. The earliest clinical applications were primarily seen in the areas of cardiothoracic imaging, neuroradiology, and breast imaging [3], with algorithms trained to analyze radiographic or mammographic images. Clinical applications of artificial intelligence and machine learning in abdominal imaging have been slower to develop, potentially due to the large number of organs/systems evaluated, reliance on multiple cross-sectional modalities, and variety of possible pathologies. However, recent years have seen the increasing development and release of algorithms for abdominal imagers.

In this manuscript, we provide an overview of artificial intelligence and machine learning techniques, review major functions that AI algorithms can perform, introduce current and potential future applications of AI in abdominal imaging, outline pathways by which AI algorithms can receive FDA approval, and explore some of the challenges faced when implementing AI in clinical practice.

## 2. Materials and Methods

For this review, a literature search was conducted using a combination of Ovid Medline, PubMed, and Google Scholar, highlighting articles from 2017–2022. Key terms included “clinical applications”, “artificial intelligence”, “machine learning”, “radiomics”, “texture analysis”, “incidental findings”, “segmentation”, “detection”, “abdominal CT”, “abdominal MR”, “radiology”, “body imaging”, and “abdominal imaging”. Non-English language results were excluded. Titles and abstracts were reviewed and selected based on their relevance by authors, all of whom are fellowship-trained in abdominal imaging with 3, 7, and 15 years of post-certification experience.

An assessment of algorithms cleared by the United States Food and Drug Administration (FDA) was also conducted at the time of manuscript preparation (April 2023). The AI Central website hosted by the American College of Radiology Data Science Institute was consulted [4], in addition to the FDA registry and non-FDA publicly available resources listed on the FDA website for Artificial Intelligence and Machine Learning (AI/ML)-Enabled Medical Devices [5]. The AI for Radiology website provided an additional resource of interest for commercially available AI algorithms in Europe, although not all algorithms listed there are approved for clinical use in the United States [6].

At the time of manuscript preparation, a total of 34 FDA-cleared SaMD products were identified within abdominal imaging, with some software having been cleared for use with more than one modality. Of these, 13 were for use with computed tomography (CT), 19 for magnetic resonance imaging (MRI), 0 for positron emission tomography (PET), 2 for ultrasound (US), and 3 for radiography (XR) or mammography. Three were for use in adult or pediatric patients, and thirty-one were cleared for use only in adult patients.

Due to the wide range of possible uses of AI and machine learning, this manuscript focuses on clinical applications that may be of interest to abdominal radiologists in the United States. “Upstream” applications spanning multiple radiologic divisions like CT de-noising, 3D printing, augmented viewing software, and applications in vascular imaging are not specifically addressed in this review.

## 3. Discussion

### 3.1. Terminology and Definitions

A basic understanding of the meaning, intersections, and divergence of the terms ‘artificial intelligence’, ‘machine learning’, and ‘deep learning’ is required to explore the landscape of SaMD in abdominal imaging. 

Artificial intelligence is the umbrella term used to encompass the development of systems capable of simulating human intelligence through learning and problem-solving. There are multiple methods that can be categorized as artificial intelligence [1,7].

Formal methods are one of the earliest techniques used to create artificial intelligence algorithms. Rather than using large datasets, formal methods use rigorous mathematical techniques to explicitly program systems in a rules-based manner, allowing responses to inputs or queries [8].

Machine learning is a subset of artificial intelligence in which systems are trained using large datasets to recognize patterns and subsequently respond to queries based on the training in relevant domains. In machine learning, most training datasets are structured, well-organized, and annotated by a ‘gold standard’ frequently requiring substantial human input. After training on these large datasets, algorithms are then able to independently respond to test datasets based upon feature mapping and statistical modeling ‘learned’ from the training datasets [1,7]. 

Deep learning is a subset of machine learning that utilizes layered ‘neural networks’, which allows unsupervised learning by the system using unorganized and unstructured data. The heuristics used by the system to develop responses to inputs and queries are independently developed by the system’s neural networks. Unlike classical machine learning, deep learning does not require substantial human input or supervision [1,7].

### 3.2. Organ Segmentation

Segmentation is defined as dividing a whole into parts or sections, which can have many applications in abdominal radiology. Much of an abdominal radiologist’s time is spent differentiating normal from abnormal tissues, identifying the organ from which an abnormality arises, or measuring boundaries and sizes. Segmentation, whether outlining organs or lesions, volumetrically tracking lesions over time, or providing an objective assessment of disease severity, was one of the first applications of AI in radiologic imaging [9]. Although precise, manual segmentation may be tedious and time-consuming. AI algorithms can expedite segmentation, either requiring no human input (automatic segmentation) or requiring limited human input during the pre-processing and/or post-processing phases (semi-automatic segmentation [10]).

To gauge the performance of a segmentation algorithm, a statistical test known as a Sørensen–Dice index or Dice similarity coefficient (DSC) is often used to compare the similarity of an automated segmentation with the “ground truth” based on manual segmentation performed by a radiologist or other trained individual, with higher DSCs indicating better performance. Segmentation is typically most accurate in organs that are discrete with less anatomical variability or abutment of surrounding structures.

Several studies have shown high accuracy of liver segmentation by AI algorithms with corresponding high Dice scores above 0.9 [11]. Many applications exist for liver segmentation, including the delineation of Couinaud liver segments, evaluation of hepatic lobar volumes before a hepatic resection or split-liver transplant donation, or assessment of liver fat fraction or iron concentration, among others. Segmentation of hepatobiliary substructures like the biliary tree, hepatic vasculature, and gallbladder has also been explored more recently [12,13].

Segmentation of genitourinary structures is also generally accurate, with reported DSCs for segmentation of the kidneys, bladder, and prostate reported to be above 0.9 [14,15,16]. Clinical uses include many oncologic applications before partial or radical nephrectomy, calculation of prostate-specific antigen (PSA) density, radiation therapy planning for prostate cancer, or partial cystectomy. Automated segmentation may also allow chronic disease processes to be more easily tracked, for example, when evaluating the total kidney volume in patients with autosomal dominant polycystic kidney disease [15].

The gastrointestinal system is challenging to segment due to the anatomical variability, complex configuration, abutment of other organs, and close apposition of discontinuous bowel loops to one another. Nonetheless, segmentation of the bowel has been pursued, with higher Dice scores found for rectum (DSCs > 0.85) [17,18,19] and colon (DSCs > 0.80) [20] than for small bowel (DSCs > 0.65) [20,21,22]. The clinical utility of segmenting the bowel includes several oncologic applications, like pre-radiation planning before pelvic radiation therapy. Other uses, like the detection and classification of inflammatory bowel disease, mucosal lesions, or bowel obstruction, have also been explored [23].

Other miscellaneous organs with high Dice scores following segmentation (DSCs~0.95) include the spleen [24], visceral or body wall adipose [25], and muscle tissue [26]. There are fewer clinical applications for splenic segmentation, although volumetric analysis of spleen size over time could be helpful when tracking disease processes that can cause splenomegaly. While not commonly performed by radiologists, automated segmentation of adipose or muscle tissue allows for opportunistic screening, as discussed below.

### 3.3. Lesion Detection

Radiologists assess and analyze images to identify, detect, and characterize disease processes. Highly sensitive early and reliable detection of lesions can play a critical role in the treatment plan for patients; for example, liver metastasis detection is paramount in emerging innovative surgical strategies for minimally invasive simultaneous resections of synchronous liver metastases in primary colorectal cancer [27]. AI has proven capable of differentiating normal and abnormal imaging findings based on algorithmic statistical models and can often provide a quantitative assessment of disease [28]. In the last decade, DL models have shown a substantial impact on the detection, segmentation, and classification of disease processes on imaging [29,30]. DL methods have been successfully used in the detection and classification of malignant lesions in various organs and imaging modalities, such as thyroid nodules on US, pulmonary nodules on CT, breast lesions on mammograms and ultrasound, pancreatic cancer on CT, and prostate cancers on MRI [28,31,32,33].

Chen and Wu et al. showed that a deep learning-based CAD tool could differentiate CT studies with and without pancreatic cancer with a sensitivity of 89.7% and a specificity of 92.8% [32]. AI-based tools have been used in segmentation, automated detection, and risk stratification with a similar area under the receiver operating characteristic curve (AUC) to expert readers in identifying clinically significant prostate cancer (81.9% expert reader vs. 83.2% CAD) [33,34]. Additionally, CAD was shown to reduce reading time in detecting suspicious prostate cancer lesions on multiparametric MRI to 2.7 min compared to 3.5 min for experienced readers and 6.7 min for moderately experienced readers [35]. Deep learning algorithms such as U-Net and fully conventional networks have been used in liver tumor detection, with segmentation-based CNN increasing the new liver tumor detection rate from 72% to 86% [36,37].

### 3.4. Detection of Incidental Findings

Incidental findings are encountered daily during the interpretation of abdominal imaging studies, particularly CT and MRI. Although many potential incidental findings exist within the abdomen and pelvis (e.g., cystic adnexal lesions, renal lesions, or adrenal lesions), there are also incidental findings that may be clinically important but originate within other organ systems included in the field of view—for example, incidental pulmonary emboli at the lung bases on a CT of the abdomen and pelvis. Due to the potential for these findings to be missed [38] and the possible need for urgent treatment or other follow-up, routine analysis of these areas by AI algorithms may be helpful to abdominal radiologists.

At the time of manuscript preparation, commercially available AI algorithms directed at incidental findings included the detection of incidental pulmonary emboli at the lung bases, free intraperitoneal air, vertebral compression fractures, or aortic dissection. Additional investigative work has also been directed toward the automated detection of various findings, including free fluid or focal inflammation (fat stranding) [39], cystic pancreatic lesions [40], vertebral body fractures [41,42], and IVC filters on radiographs [43], among others. While there is some overlap between detecting incidental findings and intentionally using a CT examination for opportunistic screening, automated acquisition of metrics on body composition, osteopenia, and others is further discussed below.

### 3.5. Characterization of Findings

While the detection of relevant imaging findings was a primary focus of early artificial intelligence models in radiology, there has been increasing effort to develop AI algorithms capable of not just detecting but also characterizing imaging findings. AI-assisted characterization has the potential to improve radiologist accuracy, throughput, and efficiency. 

Most work in this space to date has focused on developing AI capable of differentiating benign from malignant lesions; however, with increasing investment in radiomic techniques like those discussed below, tumor subtype and phenotype characterization is emerging as an additional area of research interest. While there is only a single FDA-approved algorithm designed to perform a lesion characterization function (ProstatID, BotImage, Omaha, NE, USA), there has been ample published research on potential abdominal imaging applications of AI-assisted lesions characterization in liver, biliary, kidney, adrenal glands, ovaries, and prostate.

Multiple algorithms have been developed to characterize focal liver lesions as benign or malignant on US and CT, showing an AUC comparable to radiologist performance [44,45,46]. Other research focused on the automated differentiation of hepatocellular carcinoma from other malignant tumors, such as intrahepatic cholangiocarcinoma [47,48]. Yin et al. developed a deep learning convoluted neural network (CNN) capable of distinguishing gallbladder cancer from benign gallbladder diseases on CT (AUC, 0.81; 95% CI 0.71–0.92) [49]. Another algorithm was developed by Nikpanah et al. to distinguish clear cell renal cancer from oncocytoma, with the algorithm outperforming expert radiologists included in the study (AI performance: AUC 0.81, PPV 0.78, and NPV 0.86) [50]. There have been at least three algorithms developed to date for adrenal lesion characterization, again with performance similar to or better than radiologists in published studies [51]. There have been successful proof-of-concept investigations using texture-based ML to distinguish malignant from benign ovarian lesions at CT [52]. Algorithms have also been developed to distinguish low- and high-aggressivity prostate cancer on multiparametric MRI with AUC in the range of 0.81 [53,54].

### 3.6. Opportunistic Screening

Opportunistic screening is collecting and evaluating imaging data for purposes beyond that of the primary clinical indication for the study and using imaging-based biomarkers to identify and predict individuals at risk of potential adverse clinical outcomes [55]. Visceral adipose tissue and subcutaneous adipose tissue are associated with changes in cardiovascular disease risk factors [56]. Pickhardt et al. showed that DL and feature-based automated extraction of CT biomarkers outperformed a principal clinical parameter of body mass index (BMI) in predicting major cardiovascular events in an asymptomatic outpatient cohort of 9223 adults undergoing low-dose CT screening for colorectal cancers. In this study, automated algorithms were used to quantify five parameters: aortic calcification, muscle density, visceral/subcutaneous fat ratio, liver density, and vertebral density [55]. In another study, a multivariable support vector machine model using the CT attenuation of multiple bones was used to predict osteoporosis/osteopenia [57]. AI-based opportunistic assessment of CT studies for hepatic parenchymal attenuation, surface nodularity, and volumetry has shown the feasibility of assessing fat and iron overload as well as quantitative evaluation of hepatomegaly [58,59,60]. Progressive sarcopenia (loss of muscle mass, function, or quality) has been shown to negatively predict overall and progression-free survival in colorectal cancer patients undergoing serial CTs [61] and is another feature that can be reliably extracted from images by trained AI algorithms [62].

### 3.7. Radiomics, Texture Analysis, and Quantification

Radiomics is the extraction of quantitative imaging features at the cellular or tissue level that are beyond visual perception and correlating the features to the ground truth or a specific endpoint to improve diagnostic and prognostic accuracy [63,64]. The major steps in radiomics include image acquisition, segmentation, texture feature extraction, data analysis, prediction model construction, and validation [65]. The texture feature analysis can be classified into shape-based features, histogram features, grey-level co-occurrence matrix-based features, grey-level run-length matrix-based features, and other higher-order statistical features [64]. Various studies have shown the potential of CT- and MR-based radiomics models in diagnosis, differentiation, treatment selection, and prognostic outcomes in hepatocellular carcinoma [66,67,68]. DL-based radiomic MRI features, along with clinical characteristics, have shown an accuracy of 84% and sensitivity of 94% in the preoperative prediction of tumor deposits in patients with rectal cancer [69]. CT-based radiomic texture features have shown good performance in grading and differentiating renal tumor subtypes [70]. Mukherjee et al. showed the ability of radiomics-based ML models to detect pancreatic ductal adenocarcinoma on CT imaging at a substantial lead time (median 398 days) before the clinical diagnosis [71]. Radiogenomics is another evolving area in oncologic imaging where qualitative and quantitative imaging features are extracted and correlated with the genetic profile of the tissue to generate prediction models for disease outcomes [72,73]. Both radiomics and radiogenomics have shown promising results in predicting treatment response, nodal status, and metastasis in colorectal cancer, as well as the potential for KRAS mutations [69,74,75,76]. 

While radiomics may improve future prognostication based on tumor characteristics, at a practical level, simply improving radiologists’ accuracy in identifying metastatic lesions is arguably one of the most clinically promising applications of AI lesion characterization, given the impact that metastatic disease evaluation can have prognostication. For example, Bian et al. have developed an algorithm designed to predict lymph node metastases in patients with pancreatic adenocarcinoma. In their study, the algorithm showed superior performance (AUC, 0.92; 95% CI 0.88–0.96) in predicting lymph node metastases compared to radiologists (AUC, 0.58; 95% CI 0.53–0.63) [77]. Future AI research focused on improving the accuracy, sensitivity, and specificity of staging for abdominal oncologic processes is likely to yield additional prognostication benefits.

### 3.8. FDA Approval and 510(k) Premarket Notification

The United States Food and Drug Administration (FDA) plays a critical role in ensuring the safety and effectiveness of medical products. Receiving clearance from the FDA is a key step for device manufacturers before legally marketing medical devices in the United States. Although a full description of the application, steps, and required documents is outside the scope of this clinical review, understanding certain details may be beneficial to radiologists as they approach vendors and explore available algorithms.

After being outlined in a 2019 discussion paper [78] and modified based on feedback, a plan entitled the “Artificial Intelligence and Machine Learning (AI/ML) Software as a Medical Device Action Plan” [79] was created in 2021. Under this plan, medical products that rely on AI algorithms to treat, diagnose, cure, mitigate, or prevent disease or other conditions are regulated (undergo “premarket review”) as “software as a medical device” (SaMD) using one of three pathways, each with specific data requirements [79]: premarket notification also known as 510(k) [80], premarket approval [81], and de novo classification [82]. The level of impact and risk on patients helps determine which review pathway a product should follow [83,84]. Many AI algorithms in radiology obtain approval via the premarket notification/510(k) pathway, which requires manufacturers to demonstrate that their product is “substantially equivalent” to current legally marketed devices [80]. As some AI algorithms are “unlocked” and can learn and change over time, additional review may be required according to a “predetermined change control plan” in which the ways and areas of planned AI learning/modification are outlined by device manufacturers [79].

### 3.9. Challenges

#### 3.9.1. Clinical Translation

There are many barriers, pitfalls, and challenges to the clinical translation of artificial intelligence algorithms under development. Some of these are challenges faced by all translational research endeavors; however, we will focus our discussion on barriers unique to artificial intelligence applications.

Generalizability has been an insurmountable challenge for many algorithms, making the leap from the computer science lab to clinical application. The training, validation, and even test data for algorithms shown to have spectacular performance in research studies often underestimate the variability of real-world data. It is estimated that <10% of AI models described in the literature are tested on external data [85]. Variability in imaging modality vendor, imaging parameters or protocol, and patient-related factors have all been shown to have deleterious effects on the performance statistics of artificial intelligence algorithms [86]. The challenges of generalizability are most pronounced in classical machine learning applications; however, the same challenges have been seen to affect deep learning techniques [86]. External validation partnerships between labs, increased sharing of de-identified datasets and algorithms, and development of centralized aggregate ‘real life’ clinical test data could help address generalizability concerns.

A critical consideration related to generalizability is the potential for bias introduced into artificial intelligence models, whether this occurs unintentionally in the construct of training datasets or if it develops ‘organically’ in the deep learning process of layered neural networks. Research has shown that artificial intelligence algorithms are capable of bias and are at risk of perpetuating discrimination and/or healthcare disparities if not actively mitigated [87]. Given challenges in mitigating bias in US healthcare regardless of the use of artificial intelligence, special attention to this concern is warranted as artificial intelligence algorithms are developed and deployed into clinical practice. Research specifically addressing the risks and implications of unintended bias in AI and how to avoid and mitigate these biases in algorithms is desperately needed. 

Automation bias is another pitfall cited as a major potential problem if artificial intelligence is widely adopted into clinical practice. Automation bias refers to the propensity for humans to favor suggestions from automated decision-making systems, often while ignoring sound, contradictory, non-automated information. According to researchers, there is a human expectation that artificial intelligence performs at perfect or near-perfect levels; however, human beings are likely to judge failures in artificial intelligence less harshly than the failures of humans [88,89]. These heuristics contribute to automation bias. The dangers of automation bias should encourage clinical practices to adopt artificial intelligence into their workflows to develop automation-bias mitigation strategies. Unfortunately, this is currently an area of investigation with significant unmet needs.

A unique challenge in deep learning algorithms is that the decision-making process of the system is typically opaque. Research has shown the public is overall distrusting of AI technology, with a reported 63% of citizens from five countries being unwilling to or ambivalent about trusting AI in healthcare [90]. The opaque ‘black-box’ nature of deep learning neural networks is a particularly challenging concept to overcome in building trust in artificial intelligence technology in the public sphere [91]. Consumers may demand increasingly stringent regulatory standards for the review and approval of artificial intelligence technology. Issues related to accountability, culpability, fairness, and nondiscrimination will be equally important to the public as algorithm accuracy and performance.

#### 3.9.2. Governance

The landscape of AI governance is likely to continue to evolve significantly over the next decade. Unlike governance for prescription drugs or medical devices, governance of artificial intelligence systems cannot be simply satisfied through the initial FDA evaluation and approval. Outside of the unique governance challenges related to the black-box nature of many deep learning techniques, artificial intelligence algorithms exhibit a propensity for dataset shift, resulting in a need for ongoing evaluation and oversight. Data shift occurs when training and test distributions differ. Triggers for data shift can include but are not limited to (a) new or different data acquisition techniques upstream of the model, (b) changes in IT practices upstream from the model (e.g., data structuring and naming conventions), (c) new software and IT infrastructure on which the model interacts with, and (d) changing population characteristics or demographics [92]. 

In addition, governance of AI requires consideration of privacy issues as they relate to the transmission, processing, and storage of personal data. In the European Union, the General Data Protection Regulation (GDPR) became law in 2018, defining individuals’ rights in a digital age and outlining methods to ensure compliance [93]. In the United States, medical data regulation is addressed by the Health Insurance Portability and Accountability Act (HIPAA).

It is likely that optimal models of governance for artificial intelligence in healthcare will ultimately require cooperation and shared responsibility between governmental/regulatory bodies and clinical practices deploying these systems.

#### 3.9.3. Cost

The costs to implement artificial intelligence may also create a barrier to bringing algorithms into real-world clinical workflows, particularly in the private practice setting where research and educational uses may not help to justify the costs of ownership. Costs exist in each step of the purchasing and installation process, from time spent by project managers and IT professionals to create and review a request for proposals (RFP), negotiation with preferred vendors, expenses to set up any necessary servers or build interfaces with the radiology information system (RIS) or picture archiving and communication system (PACS), operating expenses for utilizing the algorithm (most commonly an annual subscription or per-case fee rather than a single capital purchase [94]), and costs to monitor and ensure that hardware and software packages are continuing to perform optimally.

Under the predominantly fee-for-service model in the United States, few avenues currently exist to directly generate revenue using AI algorithms, as most AI analysis does not constitute a billable service. Instead, savings are often realized via improvements in efficiency, reduction in missed pathology and the potential for patient harm, or reduction in tedious work for radiologists, technologists, and other radiology department personnel. Notably, some inroads toward direct reimbursement have been made with the United States Centers for Medicare and Medicaid Services (CMS) as they consider the potential impacts that AI may have on the quality of patient care. CMS has recently developed the “New Technology Add-on Payment” (NTAP), which can help share the cost of new technologies between CMS and hospitals. Under this pathway, an example of an early—albeit currently temporary—success story for radiologists is the potential to obtain payment from CMS when an AI algorithm is used during the diagnosis and treatment of large vessel occlusion [95].

#### 3.9.4. Risk

As AI algorithms are tasked with more advanced processes (e.g., lesion characterization or opportunistic screening), many ethical and liability questions arise. In part, these concerns may also be driven by the ‘black-box’ nature of an AI algorithm, which outputs a decision and potentially a level of confidence but offers only limited ability to explore the reasoning behind the output. Additionally, when an adverse outcome like a missed diagnosis occurs, there are multiple involved parties, which may include the algorithm vendor, radiologist, or governance committee monitoring algorithm performance. At present, the radiologist bears the responsibility for rendering a final opinion on a radiologic study, regardless of what the AI output is. However, automation bias (as discussed above) may cloud judgment. If diagnostic physicians over-rely on AI outputs, there is a risk that diagnostic accuracy could decrease and result in worsened patient care in cases of AI algorithm failure [91,96,97,98,99].

The distribution of risk may also be more complex in opportunistic screening—for example, if visceral fat is automatically segmented, quantified, and recorded in the radiology report or electronic medical record, who is responsible for reviewing and making medical decisions based on the potential risk-stratification data imparted? Outputs from algorithms may reach much farther than the radiology department alone, and it will be important to hold conversations with all involved parties as algorithms are implemented within a practice or health system. 

Other risk-related topics discussed in the recent literature include data security, given the large volume of patient information needed to drive AI algorithms [99,100], the potential for malicious misuse of AI to create or alter images [101,102], and the potential for AI to impart bias based on race, gender, or other factors [87].

## 4. Conclusions

While we have already witnessed the explosive growth of artificial intelligence applications in radiology over the past decade, it is likely that we have only seen the tip of the iceberg. There is still considerable room for growth in artificial intelligence applications in radiology, particularly in abdominal imaging. At the time of manuscript preparation, there were 34 FDA-approved algorithms with applications applicable to abdominal imaging; however, there have been hundreds of research studies with promising algorithms published in just the past five years.

Advances in segmentation and lesion detection have paved the way for more advanced lesion-characterization and radiomics applications of artificial intelligence to be explored. Incidental finding detection and opportunistic screening represent two intriguing areas of algorithm development with potential benefits to all radiologists, regardless of specialty. Algorithms aimed at improving disease prognostication have the potential to be incredibly impactful in clinical practice, and we are likely to see increased investment in this space in the coming years.

The FDA approval process and post-approval governance of artificial intelligence algorithms will continue to evolve as more SaMD comes to market. The myriad barriers, challenges, and pitfalls in AI algorithm development will inform this evolution as researchers, regulatory bodies, and clinical practices work together to address concerns related to the generalizability of algorithms, bias/discrimination in algorithms, and human–machine interaction factors. Ultimately, innovation will be driven by reform in reimbursement and the proliferation of legal precedent in the space as artificial intelligence becomes a part of our daily practice environment.
